# Social Experience-Dependent Myelination: An Implication for Psychiatric Disorders

**DOI:** 10.1155/2015/465345

**Published:** 2015-05-19

**Authors:** Michihiro Toritsuka, Manabu Makinodan, Toshifumi Kishimoto

**Affiliations:** Department of Psychiatry, Faculty of Medicine, Nara Medical University, 840 Shijo-cho Kashihara, Nara 634-8522, Japan

## Abstract

Myelination is one of the strategies to promote the conduction velocity of axons in order to adjust to evolving environment in vertebrates. It has been shown that myelin formation depends on genetic programing and experience, including multiple factors, intracellular and extracellular molecules, and neuronal activities. Recently, accumulating studies have shown that myelination in the central nervous system changes more dynamically in response to neuronal activities and experience than expected. Among experiences, social experience-dependent myelination draws attention as one of the critical pathobiologies of psychiatric disorders. In this review, we summarize the mechanisms of neuronal activity-dependent and social experience-dependent myelination and discuss the contribution of social experience-dependent myelination to the pathology of psychiatric disorders.

## 1. Introduction

Throughout evolution, the conduction velocity of neurons has improved by increasing the axon diameter in order to adjust to the changing environment. An extreme example of this is giant axons observed in squid [1–3]. However, this strategy was not feasible in larger animals such as vertebrates due to physical limitations. Instead, myelination of axons by oligodendrocytes (OLs) in the central nervous system and by Schwann cells in the peripheral nervous system led to an increase in axon conduction velocity in these animals. Myelin acts as an electrical insulator. When axons were wrapped by myelin, action potential generation would occur only at nodes of Ranvier, a gap of myelin sheath, which cut down consumption of time and energy. As a result, conduction velocity of myelinated axons was quickened up to 150 m/s, whereas that of nonmyelinated axons ranges from about 0.5 to 10 m/s. This type of neuronal propagation is called saltatory conduction [2–4].

Many different factors affect myelination, especially through the processes related to the generation, migration, and differentiation of OLs and Schwann cells. These include extracellular ligands, neuronal activity, and secreted molecules [5]. It is not always clear whether mature OLs ensheath their neighboring axons properly even if the OLs express an abundant amount of myelin basic protein (MBP), a final differentiation marker of OLs. Therefore, we should recognize that the differentiation of OLs or Schwann cells and the extent of functional myelination are two different processes. Cell state of ensheathing cells has been investigated mainly by in vitro monoculture of OLs. Some reports refer to myelination only in such monocultures; however, recent studies have revealed that myelination is controlled by axoglial interactions. For example, many axonally expressed ligands such as Jagged, PSA-NCAM, and LINGO-1 inhibit myelination [6–8]. On the other hand, neuronal activity and neuronal activity-dependent secretion of adenosine and glutamate promote myelination [9–12]. Thus it has been considered necessary to use in vivo models or to use in vitro cultures with a coculture system of neurons and ensheathing cells, to assess the process or level of myelination.

A number of studies have demonstrated that neuronal activity regulates the myelination of axons [9, 10, 13]. In addition, genetic factors observed in inherited diseases, such as metachromatic leukodystrophy and Pelizaeus-Merzbacher disease, and loss of experiences after birth, such as sensory discontinuation like prelingual deafness, have been suggested to result in hypomyelination in the human brain [14]. In rodent, sensory deprivation by trimming the whiskers has been well examined model for sensory discontinuation, and this loss of experience resulted in hypomyelination in barrel cortex [15, 16]. Mangin et al. demonstrated that glutamatergic synaptic input from thalamocortical fibers on NG2-expressing oligodendrocyte progenitor cells is necessary for proper location and proliferation of NG2 cells and for final myelination [15]. Conversely, piano practicing in childhood, a kind of abundant experience after birth, is known to lead to promoted myelination in specific brain region [17]; whether the underlying molecular mechanisms of these processes are the same or not is still unclear.

In this review, we discuss how experiences, especially social experiences after birth, affect myelination based on the concept of neuronal activity-dependent myelination and how social experience-dependent myelination is related to psychiatric disorders.

## 2. Neuronal Activity-Dependent Myelination

OLs are distinguishable from Schwann cells by their ability to myelinate more than one axon. Since the thickness of myelin from OL formations differs among axons they are thought to be local factors that determine myelin thickness. Additionally, as mentioned above, the role of myelin is to facilitate conduction velocity of axons; therefore, it is reasonable to assume that the more active axons would have thicker myelin sheaths. For example, Demerens et al. demonstrated that myelination was reduced when neuronal activity was inhibited by tetrodotoxin and increased by alpha-scorpion toxin [11]. Stevens et al. revealed that neuronal activity-dependent secretion of adenosine promoted OL development and subsequent myelination [12], and Wake et al. reported neuronal activity-dependent myelination by using a coculture system of dorsal root ganglion (DRG) neurons and oligodendrocytes [10]. In the Wake et al. study, pretreatment of DRG neurons with botulinum toxin A (BnTX), which inhibits neuronal activity, resulted in suppressed myelination in coculture; this pretreatment also inhibited responses of oligodendrocyte precursor cells to Ca++ influx induced by DRG neuron activation. Antagonists of NMDA or mGluR receptors had the same effects as BnTX. Furthermore, both BnTX and glutamate receptor antagonists inhibited MBP expression in OLs. These results suggest that neuronal activity-dependent release of glutamate stimulates cells of oligodendrocyte lineage to increase Ca++ influx; these stimulated cells in turn promote the myelination of axons. This neuronal activity-dependent myelination was also demonstrated in another study using optogenetic systems [13]. Optogenetic stimulation of projection neurons in the premotor cortex resulted in increased thickness of the myelin sheath of several axon bundles within the premotor cortical circuit, from the deep layers of premotor cortex to the subcortical projections.

## 3. Myelination Promoting Factors NRG1 and BDNF

Numerous molecules have been reported as stimulants for myelination, including PDGF-A, FGF-2, IGF-1, NT-3, and CNTF secreted from astrocytes [18–21], and LIF also secreted from astrocyte stimulated by axonal release of ATP [22]. Of course these secreted molecules are indispensable for sufficient development of myelination, but the correlation between neuronal activity and secretion of these molecules is unclear. Here discussing direct interaction between neurons and ensheathing glial cells, we will focus on two neuronal activity-dependent neurotrophins, neuregulin-1 (NRG1) and brain-derived neurotrophic factor (BDNF). NRG1 is known to have many splice variants, among which types I, II, and IV show neuronal activity-dependent expression [23]. NRG1 plays a crucial role for myelination in PNS [24], but its role in the CNS remains controversial. Several studies described the importance of NRG1 and its receptor ErbB for myelination in the CNS: studies in mice lacking ErbB2 signaling [25], type III NRG1 knockout mice [26], mice lacking OL specific NRG-ErbB signaling [27], and OL specific ErbB3 knockout mice [28] showed hypomyelination in the CNS. In contrast, NRG1 type I or type III overexpressing mice showed hypermyelination in the CNS [29]. However, Brinkmann et al. reported that it is not necessary for CNS myelination because several types of NRG1 or ErbB knockout mice showed normal myelination in their study [29]. Similarly, BDNF is secreted in an activity-dependent manner [30] and is reported in relation to myelination. BDNF knockout mice show hypomyelination [31] and BDNF promotes myelination by OLs in coculture with DRG neurons by TrkB receptor activation [32].

## 4. NRG1, BDNF, and Neuronal Activity-Dependent Myelination

**Figure 1:**
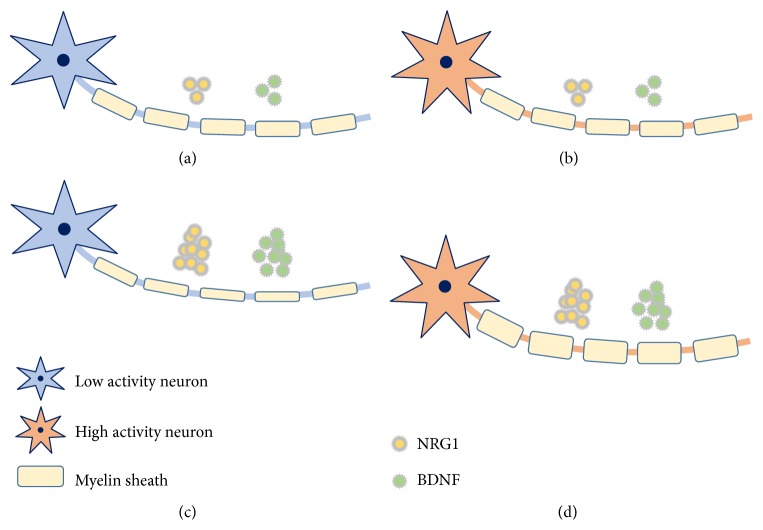


It has been reported that neuronal activity, NRG1, or BDNF each promotes myelination, as mentioned above, but whether these factors could interact or not is unknown. Lundgaard et al. reported the interesting association between NRG1 or BDNF and neuronal activity-dependent myelination [33]. They utilized a coculture system of DRG neurons and OLs in their analysis. By default, when cocultured without NRG1 or BDNF, axons of DRG neurons were myelinated independent of neuronal activity, but if cocultured with NRG1 or BDNF, OLs myelinated axons in an activity-dependent manner [33]. This result implies that the addition of NRG1 or BDNF at a proper concentration converts the mechanism of myelination by OLs from neuronal activity-independent to activity-dependent. When comparing the myelination of axons cocultured with NRG1 to those without NRG1, the former show more myelination, as described previously [26]. However, even with NRG1 added, axons cocultured with OLs in the neuronal activity-dependent mode are less myelinated if neuronal activity is blocked than axons cocultured with OLs without NRG1 in the neuronal activity independent mode (Figure 1). That is, neuronal activity causes more myelination than NRG1 in some situations. These results may account for the discrepancy in results described above regarding the relationship between myelination and NRG1 in the CNS.

## 5. Sociality and Prefrontal Cortex

Sociality (or sociability) is defined as an ability to behave by inferring the mindset, intent, and beliefs of others in the situation, in order to develop a relationship with them. Of course, among healthy subjects the extent of sociality varies among individuals; however, patients with Autism Spectrum Disorder (ASD) show a deficit in this ability, which leads to socializing difficulties in daily life. Because of this, recent studies compared patients with ASD to healthy subjects in different aspects to investigate the biological basis of sociality. From these studies, the following results were observed: healthy subjects showed activation of the medial prefrontal cortex (PFC) during fMRI or PET scans while solving the “theory of mind” task, a type of sociality postulating task [34, 35]. Additionally, during eye contact and mutual gaze kinds of nonverbal communication required for acquisition of “theory of mind,” the right inferior frontal gyrus and medial frontal cortex were activated in healthy subjects [36, 37]. Activation of the medial PFC was also induced by stimuli initiating dishonor or guilty feelings, which arise only in those having a consciousness of other's opinions or view of oneself [38]. Such frontal lobe activation observed in healthy subjects has not been shown in patients with ASD during these imaging studies; therefore there could be a correlation between sociality and frontal lobe activity.

## 6. Social Experience-Dependent Myelination in the PFC

As mentioned above, social experience activates the PFC, that is, social experience promotes neuronal activation in PFC neurons. Activation of an axon stimulates secretion of adenosine and glutamate from the neuron, which leads to activity-dependent myelination. A well-known example of loss of social experience is socially isolated individuals like orphan children born in Romania under communism. The Romanian dictator Nicolae Ceausescu carried out his policy of augmenting the population by providing scholarships to women who had more children and prohibiting contraception, abortion, and divorce. However, these policies resulted in most children being nurtured in orphanages with aversive environments due to the poor economic planning. These so-called Ceausescu's children suffered from psychiatric disorders such as attention-deficit/hyperactivity disorder (ADHD) even after being nurtured later in a loving family with adopted parents [39]. Structural MRI studies showed that the total volume of gray and white matter of these orphans was lesser and that of the amygdala was larger than normally raised children [40]. In addition, analysis by diffusion tensor imaging revealed hypomyelination of the uncinate fasciculus in the orphans, a prefrontal related neuronal pathway, as described by the lower fractional anisotropy (FA) [41]. From these studies, it became apparent that not only sensory and motional stimulation, but also the quality of social experiences alters the state of myelination. We have previously reported that social isolation in juvenile mice leads to hypomyelination of the PFC and that Neuregulin-ErbB signaling plays a crucial role in this mechanism [28]. Liu et al. also reported hypomyelination of the PFC in mice lacking social experiences possibly due to epigenetic alterations of OLs [42].

## 7. Role of Myelination

**Figure 2:**
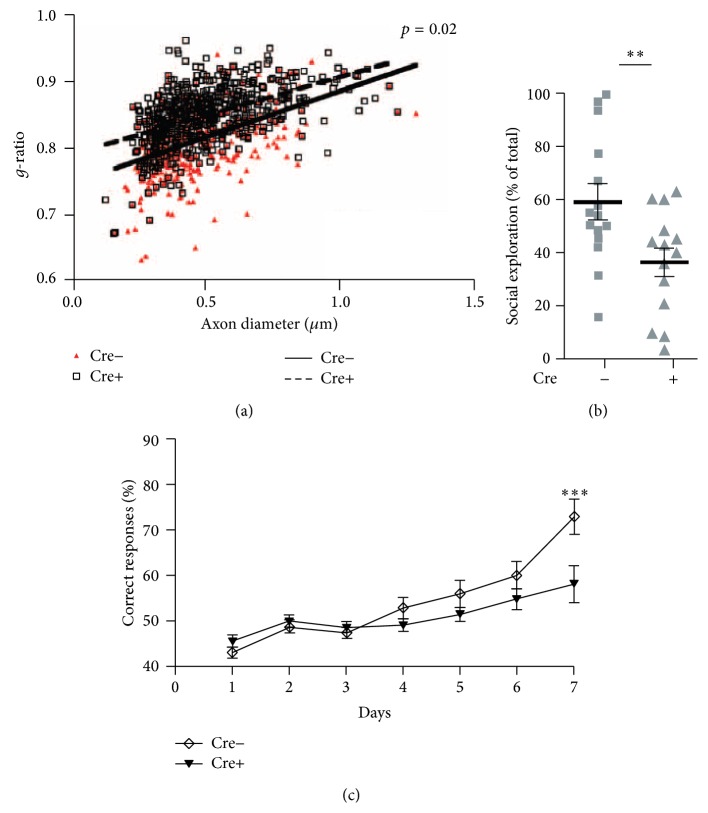
Modified from Makinodan et al., Science [28]. (a) Myelin thickness. (b) Sociality. (c) Working memory.

We have discussed the influence of social experience on myelination in previous sections; however, we could learn much more from patients of multiple sclerosis since they too show clinical symptoms related to the brain region where demyelination has occurred. For example, demyelination in the motor cortex disturbs gait, and in the hippocampus, it affects memory. We questioned how demyelination affected the PFC. We know several brain functions are dependent on the PFC, including working memory, attention, sociality, and anxiety, but there exists no report directly establishing the relationship between the myelination of the PFC and these brain functions in humans. Even in animal experiments, no report has shown such causation by analyzing the results of PFC-dependent behavioral tasks in mice or rat with PFC-specific myelination deficits. We have not clarified the cause but the correlation between myelination of the PFC and PFC-dependent brain functions by using model animals with myelination deficits not specific to the PFC: (1) mice fed with cuprizone, a type of copper chelator, for 4 weeks showed deficits of working memory and sociality with demyelination in the PFC and hippocampus [43]; (2) OL specific Neuregulin-ErbB signaling deficient mice (expressing a dominant-negative ErbB4 receptor under the control of promoter for 2′,3′-cyclic nucleotide 3′-phosphodiesterase) had defective myelination in the PFC and showed deficits of working memory and sociality (Figure 2(a)) [28]; (3) ErbB3 receptor (a type of NRG1 receptor mainly expressed on OL) knockout mice also had defective myelination in the PFC and showed deficits of working memory and sociality (Figure 2(b)) [28].

PFC myelination may be necessary for the acquisition of working memory and sociality. For example, it has been shown by McKenzie et al. that renewed myelination in white matter is necessary for proper motor function [44]. The same finding may be accurate for the PFC and PFC-dependent abilities. We have already reported that social experiences in juveniles are necessary for PFC myelination in our mouse model. Later acquisition of PFC-dependent working memory and sociality would be impaired in these mice if renewed myelination were disturbed by some factors like unsuitable genetic programing.

## 8. Conclusion

One possible mechanism how experiences affect myelination is the following: experiences drive neuronal activity in a related brain area; neuronal activity converts the mechanisms of myelination into the neuronal activity-dependent mode by promoting secretion of NRG1 and BDNF; myelination in neuronal activity-dependent mode is accelerated by additional neuronal activity. In this case, both neuronal activity and neurotrophins such as NRG1 and BDNF are necessary for proper myelination.

Social experiences, especially during the juvenile period, are one of the influential matters for the development of psychiatric disorders, in some groups of which myelination is impaired. Therefore, together with findings as described in this review, it is possible that juvenile social experience-dependent myelination occurs through the expression of well-known risk molecules for psychiatric disorders, NRG1 and BDNF, and results in the development of psychiatric disorders.
